# Inhibitory impact of A1 and A2 cow’s milk on human peripheral blood mononuclear cells identifies TOM1 as a candidate anti-inflammatory mediator

**DOI:** 10.3389/fimmu.2026.1728935

**Published:** 2026-06-10

**Authors:** Lili M. Flad, Tanja Weißer, Jonas Liedl, Felix Gard, Agnese Petrera, Christine von Toerne, Stefanie M. Hauck, Cornelia A. Deeg, Kristina J. H. Kleinwort

**Affiliations:** 1 Chair of Physiology, Department of Veterinary Sciences, Ludwig Maximilian University of Munich, Martinsried, Germany; 2 Metabolomics and Proteomics Core, Helmholtz Center Munich, German Research Center for Environmental Health, Munich, Germany

**Keywords:** A1/A2 milk fractions, ConA, immunonutrition, Olink, PBMC, proliferation, supernatant proteome, TOM1

## Abstract

**Introduction:**

Cow´s milk has long been discussed as a modulator of immune response, with a particular focus on different health effects of its β-casein variants A1 and A2. To date, the factors in milk which may be responsible for effects on the human immune system remain unknown, revealing a lack of information that warrants further investigations. We previously found that both A1 and A2 milk inhibit Concanavalin A-induced human peripheral blood mononuclear cells (PBMC) proliferation. Accordingly, this study aimed to investigate the underlying mechanisms of the inhibitory effect.

**Methods:**

In a first step, we narrowed down the size-range of possible causative factors in A1 and A2 milk samples by separating each milk type in a high (> 100 kDa; HWF) and a low molecular weight fraction (< 100 kDa, LWF) and used them in co-incubation with Concanavalin A stimulated human PBMC. In *in vitro* proliferation assays, we identified HWFs as the inhibitory fractions and analyzed the PBMC´s supernatant proteomes using high-resolution LC-MS/MS to assess changes in the cellular response to HWFs. Olink proteomics was used to asses the influence of the candidate protein on Immune-Response-Marker release of PBMC.

**Results:**

HWFs of milk exert an inhibitory effect on human PBMC. Among several candidates, Target of Myb protein 1 (TOM1) emerged as the only protein markedly increased in the supernatant proteomes after co-incubation with the HWFs of A1 and A2 milk. Further *in vitro* proliferation assays revealed an inhibitory impact of TOM1 on stimulated humane PBMC. Olink analysis of PBMC Immune-Response-Marker secretion reinforced a possible role for TOM1 as anti-inflammatory mediator in milk involved in the regulation of immune response via Interleukin-1β-associated signaling.

**Discussion:**

Our findings provide new insights into milk-induced immune regulation and identify TOM1 as a promising anti-inflammatory factor.

## 1 Introduction

There is an ongoing scientific debate about the role of cow´s milk as a dietary component, particularly regarding health effects associated with different β-casein variants. This discussion mainly focuses on differences between the A1 and the A2 variants, which are the most prevalent in European dairy cattle (1–4). These two forms differ by a single amino acid substitution at position 67 of β-casein (5, 6), caused by a point mutation in the CSN2 gene (4). This amino acid substitution is thought to render the A1 variant more susceptible to enzymatic cleavage during digestion, resulting in the increased release of β-casomorphin-7 (7). This peptide has been implicated in modulating immune responses in humans (8). Several studies have investigated the effects of β-casomorphin-7 on human peripheral blood mononuclear cells (PBMC), reporting a range of outcomes, including stimulatory, inhibitory, or, in some cases, no significant impact on cell proliferation (9–11). In contrast, studies on hydrolysates from other, uncharacterized bovine β-casein variants demonstrated an inhibitory effect on human PBMC proliferation *in vitro* (12). Similarly, we previously showed that both A1 and A2 milk also have a marked inhibitory effect on proliferation of polyclonally stimulated human PBMC (11). These findings confirm a suppressive effect of cow´s milk, regardless of the A1 or A2 β-casein variant.

Because our own prior data show that A1 and A2 milk exert comparable anti-proliferative activity on Concanavalin A (ConA)-stimulated human PBMC (11), the identity of the causative factor in milk - rather than the β-casein variant carrying it - becomes the central question. A pragmatic strategy to narrow down the search space is to fractionate native milk by molecular weight, which divides the candidate space into a high-molecular-weight compartment (intact proteins, protein complexes, casein micelles, milk fat globule membrane fragments, extracellular vesicles) and a low-molecular-weight compartment (small proteins, peptides, soluble metabolites). Testing each compartment separately on primary human immune cells makes it possible to assign the biological activity to a defined size range before any individual candidate is pursued.

With respect to likely molecular targets, several well-characterized pathways by which high-molecular-weight milk components could modulate T-cell activation are known from other systems. Components of the IL-1 family signaling axis, including the IL-1 receptor 1 (IL-1R1) and its endosomal adaptor TOLLIP, are regulated through receptor trafficking, ubiquitination and lysosomal degradation (13, 14). The NF-κB and AP-1 transcription factors downstream of IL-1R1 govern a large fraction of the pro-inflammatory cytokine program of activated T-cells (15, 16). A bovine-derived bioactive that interferes with any of these steps could therefore modulate T-cell activation without directly blocking the T-cell receptor or CD28 co-stimulation. These mechanisms motivated our choice of an unbiased supernatant-proteome analysis.

In the present study we therefore fractionated both A1 and A2 milk into a high (> 100 kDa, HWF) and a low molecular weight fraction (< 100 kDa, LWF), tested each fraction for its anti-proliferative effect with ConA-stimulated primary human PBMC, and performed an unbiased LC-MS/MS analysis of the PBMC supernatant proteome with the aim of generating mechanistic hypotheses regarding the cellular response to the anti-proliferative milk components. The functional relevance of the leading candidate identified in this screen was subsequently assessed in recombinant-protein follow-up experiments.

## 2 Materials and methods

### 2.1 A1 and A2 milk

Milk of both the A1 and A2 type was manually collected at the same day at a volume of 500 mL each from three cows per group, each homozygous for the respective A1 or A2 variant of the β-casein gene and were kept at 4 °C immediately after milking. The cows, all the same breed (Simmental), were fed an identical diet and kept under identical environmental conditions as they originated from a single herd and stable. The milk from three cows per genotype was subsequently pooled into a single genotype-level milk sample. This design was chosen to capture biological breadth across individual animals while averaging inter-individual variability at the milk-source level, thereby concentrating statistical power on the human PBMC-donor axis. As a consequence, the biological replication at the milk-source level is effectively n = 1 per genotype.

### 2.2 Milk fractions

Milk was skimmed by centrifugation (four times, 4 °C, 1000 x g, 5 min) and a 100 kDa molecular weight cut-off filter was used (three times, 4 °C, 4000 x g, 20 min; Merck Millipore, Darmstadt, Germany) to separate each milk into two fractions: the flow-through and the retentate, representing the low molecular weight (LWF) and the high molecular weight (HWF) fraction respectively. The HWF (> 100 kDa) retains intact proteins together with larger supramolecular assemblies such as casein micelles, milk fat globule membrane fragments and extracellular vesicles; the LWF (< 100 kDa) contains small proteins, peptides and soluble metabolites. No compositional profiling was performed on these fractions; the biological activity described in this work is therefore ascribed to the cumulative effect of all components within the indicated size range.

To prevent artificial enrichment of proteins due to filter-aided centrifugation, fractions were reconstituted with phosphate-buffered saline (PBS; NaCl 136.9 mM, Na2HPO4 × 2H2O 8.1 mM, KH2PO4 1.4 mM and KCl 2.6 mM; pH 7.4) to their original volumes, ensuring consistency across conditions. Fractions were not normalized to a common total-protein concentration; instead, each fraction was reconstituted in PBS to the volume of the native milk from which it was derived and subsequently added at 1% v/v to the PBMC cultures. Thus, HWF and LWF of each milk variant deliver the protein mass natively present in the respective molecular-weight range within 1% milk, preserving the physiological stoichiometry between fraction and matrix. Subsequently, the fractions of each milk were pooled according to the β-casein genotype and molecular weight, resulting in four distinct pools, each consisting of milk from three different cows: A1 > 100 kDa (A1 HWF), A1 < 100 kDa (A1 LWF), A2 > 100 kDa (A2 HWF), and A2 < 100 kDa (A2 LWF) (**Supplementary Figure 1**). After pooling, samples were divided into aliquots to avoid repeated freeze-thaw cycles and stored at −20 °C prior to use for at most one month. All four samples were treated using identical procedures, under identical storage and thawing conditions, and with identical preheating on the day of the experiment.

The fractioning into two molecular weight fractions (HWF and LWF) was based on pretests: initially, four fractions < 100 kDa (< 3 kDa, < 10 kDa, < 50 kDa and < 100 kDa) and one fraction > 100 kDa were generated. In preliminary experiments, all fractions < 100 kDa showed no significant impact (**Supplementary Figure 2**) so we focused on the comparison between the HWF and the LWF.

### 2.3 Donors and isolation of human primary PBMC

Venous whole blood donations from 15 healthy voluntary human donors were provided in sodium-heparin-coated tubes. Not all donors contributed to every assay; exactly it was n = 10 for the milk-fraction proliferation assays, n = 9 for the recombinant-TOM1 dose-response proliferation assays, n = 3 each for LC-MS/MS and Olink). All donors gave their written informed consent for inclusion before they participated in the study as described (11).

The blood was diluted 1:2 with PBS (pH 7.4) and Pancoll separation solution (PanBiotech, Aidenbach, Germany) was used for density gradient centrifugation (room temperature (RT), 500 × g, 25 min). PBMC were collected from the buffy coat and washed twice with PBS (pH 7.4; 4 °C, 800 x g, 10 min).

### 2.4 *In vitro* cell proliferation of PBMC

After resuspension in RPMI 1640 (PanBiotech) with 10% fetal calf serum (FCS; Sigma-Aldrich, Darmstadt, Germany) and 1% penicillin-streptomycin (Sigma-Aldrich), triplicates of each 2 × 10^5^ human PBMC were polyclonally stimulated with 1 μg/mL Concanavalin A (ConA; Sigma-Aldrich) and incubated in 96-well U-bottom plates (Sarstedt, Nümbrecht, Germany) at 37 °C and 5% CO2. From each donor, unstimulated PBMC served as negative controls and ConA-stimulated PBMC without co-incubation served as a set point for the polyclonal stimulation (= positive control). We assessed the effects of various milk components on PBMC proliferation (n = 10) by supplementing ConA-stimulated PBMC cultures with the previously described milk fractions at a final concentration of 1%. Additionally, we examined the effect of the protein Target of Myb protein 1 (TOM1; Biomol, Hamburg, Germany) on proliferation of PBMC (n = 9), by co-incubating ConA-stimulated PBMC with purified TOM1 at different concentrations, ranging from 0.1 ng/ml to 1000 ng/ml.

PBMC were labeled with 1 μCi [3H]-thymidine (PerkinElmer, Rodgau, Germany) per well eight hours before harvesting. Harvesting was performed after 48 hours of stimulation using a FilterMate Cell Harvester (PerkinElmer) and the matching MicroBeta filter mats (Perkin Elmer). Radioactivity per well was measured by a β-scintillation counter MicroBeta2 (PerkinElmer).

To quantify the effects of ConA on PBMC, human PBMC were stimulated with ConA in triplicates. Proliferation was quantified as counts per minute (cpm) for each well, and the cpm values within each triplicate were averaged for further calculations. To evaluate donor-specific response to ConA, the mean value of ConA-stimulated PBMC was divided by the corresponding mean value of the unstimulated negative control, allowing the determination of the fold increase in proliferation induced by polyclonal stimulation. Only donors whose PBMCs demonstrated a ≥ 15-fold increase in proliferation following ConA stimulation compared to unstimulated controls were included. This threshold was established empirically based on extensive laboratory experience and was set high to ensure inclusion of genuinely immunocompetent donors only. This led to a total number of 14 included donors, in which ConA stimulation resulted in an average 37.5-fold (SD ± 23.4) increase in proliferation compared to the unstimulated control.

To quantify the effects of milk fractions and TOM1 on proliferation of polyclonally stimulated PBMC, ConA-stimulated triplicates of each donor were supplemented simultaneously with either the previously described milk fractions or a serial dilution of the purified protein TOM1 at the indicated concentrations. The mean value of the ConA-stimulated triplicates of each donor was arbitrarily set to 100%. The mean values of the triplicates co-incubated with milk fractions or TOM1 were then normalized to this reference value for each individual donor. This approach was used to represent the effects of milk fractions or TOM1 relative to polyclonal stimulation and to enable more reliable inter-donor comparisons.

### 2.5 Polyclonal stimulation of PBMC for mass spectrometric analysis

After resuspension in serum-free RPMI 1640 (PanBiotech) with 1% penicillin-streptomycin (Sigma-Aldrich), PBMC from three donors were seeded in 6-well plates (Sarstedt) with 1 x 10^7^ cells per well. For each donor, PBMC were stimulated with 1 µg/mL ConA (Sigma-Aldrich) and co-incubated at the same time in separate wells with each of the four previously described milk fractions in a final concentration of 1% at 37 °C and 5% CO2. This resulted in four different co-incubations per donor and a total number of 12 across all three donors. Serum-free RPMI 1640 supplemented only with 1% penicillin-streptomycin was used to avoid interference of exogenous proteins with the detection of low-abundance supernatant proteomes components. No FCS or artificial FCS substitutes (such as protein-defined supplements containing albumin, insulin, transferrin, selenium or lipids) were added, as these would mask secreted proteins and bias downstream proteomic analysis. The viability of the cells and monitoring of cell lysis were closely analyzed (light microscopy, trypan blue staining, flow cytometric Annexin V/PI-staining) during the experimental period. Following 48 hours of incubation, supernatants were collected for mass spectrometric analyses to discover the cellular secretory response of PBMC to co-incubation with the HWFs in comparison to the LWFs at the proteomic level. Supernatant proteomes were separated from cells using centrifugation (4 °C, 800 x g, 10 min) and were purified with another centrifugation step (4 °C, 1000 x g, 10 min). One mL supernatant of each co-incubation per donor was stored in low-bind tubes (Sarstedt) at -80 °C until mass spectrometric analyses.

### 2.6 Sample digestion for mass spectrometric analysis

Supernatants were digested with Lys-C and trypsin following a modified filter-aided sample preparation, as previously described (17, 18). Briefly, trichloroacetic acid (Sigma-Aldrich) was added to a final concentration of 25% to supernatants (4 °C, 10 min) and protein pellets were generated using centrifugation (RT, 14,000 x g, 5 min) and discarding the supernatants afterwards. The protein pellets were washed three times with ice cold acetone, incubated with Ammonium Bicarbonate buffer (50 mM NH3HCO3 (Sigma-Aldrich) diluted in HPLC-grade water; RT, 10 min) and 20 mM dithiothreitol (SERVA, Heidelberg, Germany) was added for 30 minutes at 60 °C. After cooling down to RT, samples were incubated with 40 mM iodoacetamide (Merck Millipore) in the dark for 30 minutes and free iodoacetamide was quenched with 40 mM dithiothreitol. Proteins were subjected to proteolysis for 2 hours at RT by adding 0.1 µg Lys-C followed by an addition of 1 µg trypsin overnight at 37 °C. Peptides were purified as previously described (19).

### 2.7 Mass spectrometric analysis

Acidified eluted peptides were analyzed on a Q-Exactive HF-X mass spectrometer (Thermo Fisher Scientific, Waltham, MA, USA) online coupled to an Ultimate 3000 RSLC nano-HPLC (Thermo Fisher Scientific). Samples were automatically injected and loaded onto the C18 trap cartridge, eluted after 5 minutes and separated on the C18 analytical column (Acquity UPLC M-Class HSS T3 Column, 1.8 μm, 75 μm x 250 mm; Waters, Rydalmere, NSW, Australia) by a 90 minute non-linear acetonitrile gradient at a flow rate of 250 nL/min. MS spectra were recorded at a resolution of 60,000 with an AGC target of 3 x 10^6^ and a maximum injection time of 30 ms from 300 to 1500 m/z. From the MS scan, the 15 most abundant peptide ions were selected for fragmentation via HCD with a normalized collision energy of 28, an isolation window of 1.6 m/z, and a dynamic exclusion of 30 s. MS/MS spectra were recorded at a resolution of 15,000 with an AGC target of 1 x 10^5^ and a maximum injection time of 50 ms. Unassigned charges, and charges of + 1 and > 8 were excluded from precursor selection.

### 2.8 Protein identification, MS label-free quantification

Peptide and protein identification were carried out using Proteome Discoverer 2.5 (Thermo Fisher Scientific, Waltham, MA, USA) via a Sequest HT database search against Swissprot human database (Release 2020_02, 20432 sequences) and Ensemble Cow (Release 2014_02 UMD3.1; 22118 sequences). Full tryptic specificity was applied, allowing one missed cleavage. The precursor mass tolerance was set to 10 ppm, and the fragment mass tolerance was set to 0.02 Da. Carbamidomethylation of cysteine was set as a static modification, while deamidation of asparagine and glutamine, methionine oxidation, and methionine loss with N-terminal acetylation were defined as dynamic modifications.

Percolator validated peptide spectrum matches and peptides, accepting only the top-scoring hit for each spectrum with false discovery rates < 1% and posterior error probability < 0.05. The Sequest HT Xcorr filter threshold was set to 1.6, thereby restricting further analysis to high-confidence peptide matches only. Protein inference was performed using the parsimony principle, which assigns peptides only to the minimal set of proteins explaining the data. To address potential species-overlap between human and bovine sequences, protein inference was additionally repeated without strict parsimony. This analysis ensured that all possible assignments in the combined database were retained and that human proteins of interest, such as TOM1, were consistently supported by unique peptides. The resulting datasets are provided **in Supplementary Tables 1**, **S2**, and **S3**.

Quantification was based on the abundance values for the top three unique peptides, which were normalized against total abundance to account for sample loading errors. Data were exported to Perseus software (1.6.14.0) (20). After log2 transformation low abundance imputation was performed according to manufacturer’s settings. Limma-batch correction on replicates as confounding factors was performed and was followed by Students t-test with Benjamini Hochberg correction as statistical analysis.

### 2.9 Statistical analysis

To determine whether the proliferation data followed a Gaussian distribution, the Shapiro–Wilk test was applied. As all data were normally distributed (Shapiro–Wilk test with p > 0.05), paired t-tests were then employed for statistical analysis between samples. For statistical analysis of proliferation samples comparing with ConA control condition, Wilcoxon signed-rank tests were performed, as the control condition (ConA) showed no variance, because of setting the control values to 100. Statistical significance was set at p ≤ 0.05. For statistical analysis and to visualize the proliferation data, GraphPad Prism (version 5.04 for Windows, GraphPad Software, San Diego, CA, USA) was used.

The aim of the proteomic data analysis was to identify proteins with altered abundance in the supernatant proteome of polyclonally stimulated PBMC following incubation with A1 and A2 HWF in comparison to the LWF. For co-incubation with each milk variant (A1 and A2), protein abundances in supernatants after the HWF co-incubation were compared directly to the corresponding abundances after co-incubation with the LWF from the same variant. The analysis was therefore directed at detecting proteins that were at least two-fold more abundant in supernatants with co-incubation of the HWF relative to the LWF incubation of the same milk variant. All fold changes presented in this work refer exclusively to these comparisons unless stated otherwise. Additionally, to ensure detection reliability, only proteins that were detected in more than six out of twelve supernatants and that were identified by at least two unique peptides, were included in further analysis. The applied filtering criteria were chosen to prioritize reliable, robust candidates for functional follow-up studies. This comparative strategy allowed a focused identification of proteins increased due to the co-incubation of ConA-stimulated PBMC with the A1 and A2 HWF.

The mass spectrometry proteomics data generated for this study were deposited to the ProteomeXchange Consortium via the PRIDE (21) partner repository (https://www.ebi.ac.uk/pride/archive) with the dataset identifier PXD066232. Reviewers can access the dataset at https://www.ebi.ac.uk/pride/(accessed on 17 July 2025) using the following account details: Username: reviewer_pxd066232@ebi.ac.u Password: VaUtGu57Q31j.

To provide an overview and to clearly visualize the key findings of the proteomic data according to large fold changes and high statistical significance, volcano plots were generated using GraphPad Prism (version 5.04 for Windows, GraphPad Software, San Diego, CA, USA). To separately analyze the secreted proteins with differential abundance in response to the A1 and A2 HWF, and to determine the overlap between the two co-incubations, a venn diagram was designed with InteractiVenn (https://www.interactivenn.net/index.html, accessed on 02 July 2025) (22). Protein-protein interactions of TOM1 were visualized with open-source software String version 12.0 (https://string-db.org, accessed on 11 September 2025) and information about molecular weight of proteins of interest was obtained by using open-source database Uniprot (https://www.uniprot.org, accessed on 23 September 2025) (23). To identify proteins that have previously been reported in cow´s milk we used open-source database BoMiProt2.0 (https://bomiprot.org, accessed on 12 September 2025) (24, 25). The open-access database The Human Protein Atlas (26) was used to get information about expressed proteins in human PBMC and subtypes (https://www.proteinatlas.org, accessed on 13 September 2025).

### 2.10 Olink proteomic profiling

After resuspension in serum-free RPMI 1640 (PanBiotech) with 1% penicillin-streptomycin (Sigma-Aldrich), PBMC from three donors were seeded in 48-well plates (Sarstedt) with 3 x 10^6^ cells per well. For each donor, PBMC were stimulated with 1 µg/mL ConA (Sigma-Aldrich) and co-incubated at the same time in separate wells with TOM1 (1 µg/ml; Biomol) at 37 °C and 5% CO_2_, resulting in one ConA control sample and one ConA + TOM1 sample per donor. Following 48 hours of incubation, supernatants were collected to discover the cellular secretory response of PBMC to co-incubation with TOM1. Supernatants were separated from cells using centrifugation (800 x g, 5 min) and subsequently stored at −80 °C prior to measurement.

The concentration of 44 Immune-Response-Marker in PBMC cell culture supernatants after co-incubation with ConA and TOM1 was determined using the Olink Target 48 Immune Surveillance Panel (Olink Proteomics AB, Uppsala, Sweden). In brief, samples were incubated with DNA barcode–tagged antibody pairs. Amplification and quantitation of antigen-specific DNA barcodes was then carried out by multiplex PCR on an Olink Signature Q100, and calibration was used to determine absolute cytokine concentration. Raw data are provided in **Supplementary Table 4**. For further analysis, data of the following proteins were omitted because of missing quantification in at least one sample: CCL26, IFNB1, FGF23, FGF21, IL17D, EPO, KRT18, IL31 and IFNA2. In order to analyze the secretory response of human PBMC to co-incubation with TOM1, quantified values of background (RPMI cell medium with ConA control) were subtracted from the values after TOM1-incubation (ConA + TOM1). For visualization of secretion patterns, the quantified values were transformed to arcsinh-values and heatmap was created using the R package *pheatmap*.

## 3 Results

### 3.1 High molecular weight fraction of both A1 and A2 milk inhibited proliferation of PBMC

**Figure 1:**
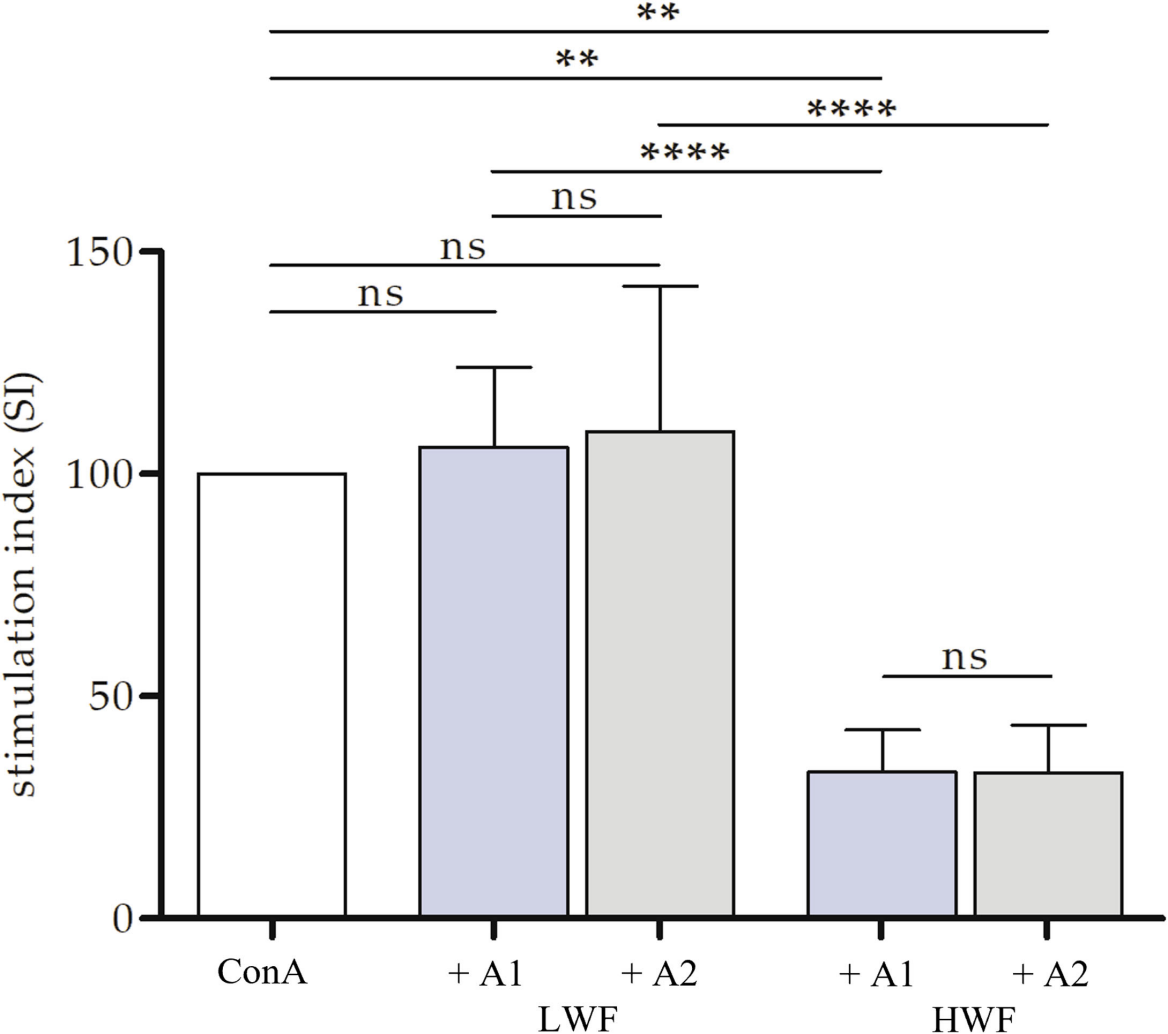
Effects of the low (LWF) and high molecular weight fraction (HWF) of A1 and A2 milk on ConA-induced proliferation of human PBMC (n = 10). Bar plots represent stimulation indices of ConA-stimulated PBMCs following incubation with the fraction above 100kDa and below 100kDa of both A1 (blue) and A2 (gray) milk at a total concentration of 1%, n = 10. ConA-stimulated PBMCs served as control and were set to 100 (white), n = 10. The Shapiro–Wilk test was used to determine Gaussian distribution. As the control condition (ConA) showed no variance because of setting to 100, comparisons involving this group were performed using the Wilcoxon signed-rank test. For all other paired comparisons, paired t-tests were applied; ns, p > 0.05; **, p < 0.005; ****, p < 0.0001.

Significant differences in the inhibition of polyclonal stimulation of human PBMC (n = 10) were observed following incubation with the HWF and the LWF of both A1 (**Figure 1**; blue columns) and A2 milk (**Figure 1**; gray columns). Both LWFs did not alter PBMC after polyclonal stimulation (**Figure 1**). In contrast, both the A1 HWF (stimulation index [SI]: 33.1 ± 9.3 SD; **, p < 0.005; **Figure 1**) and the A2 HWF (SI: 32.9 ± 10.7 SD; **, p < 0.005; **Figure 1**) significantly suppressed polyclonally stimulated proliferation. As this inhibitory effect was comparable between A1 and A2 milk, no significant differences were found between the two β-casein variants (**Figure 1**). A parallel flow cytometric Annexin V/PI-staining experiment confirmed that the inhibitory effect caused by the HWFs was not due to cell death.

Given the equal inhibitory potential of the A1 and A2 HWF, we hypothesized that the effect was mediated by a shared, yet unidentified protein present in both HWFs independent of the β-casein variant. To identify the mechanisms activated by this protein, a differential proteomic analysis was performed.

### 3.2 The supernatant proteome of human PBMC after incubation with milk fractions consists of 2640 human proteins

To elucidate the anti-proliferative effect of the HWF of both milk variants on the polyclonally stimulated human PBMC in comparison with the LWF on protein level and to get insights into the cell mechanisms induced by proteins of the proliferation-inhibiting HWF, a proteomic approach was applied. Using LC-MS/MS analysis, a total of 3199 proteins in the supernatant proteome of polyclonally stimulated PBMC after co-incubation with the HWF and the LWF of both A1 and A2 milk were detected, of which 2640 were identified as human and 559 proteins as bovine. In order to determine which proteins were more abundant in the supernatant proteome as a response of human PBMC to co-incubation with the proliferation-inhibiting HWF, the further analysis of the proteomic data was restricted to human proteins. A total of 2129 proteins met the previously described criteria and were included in further analysis.

### 3.3 Differentially abundant proteins in the PBMC supernatant proteome after treatment with the HWF were identified

**Figure 2:**
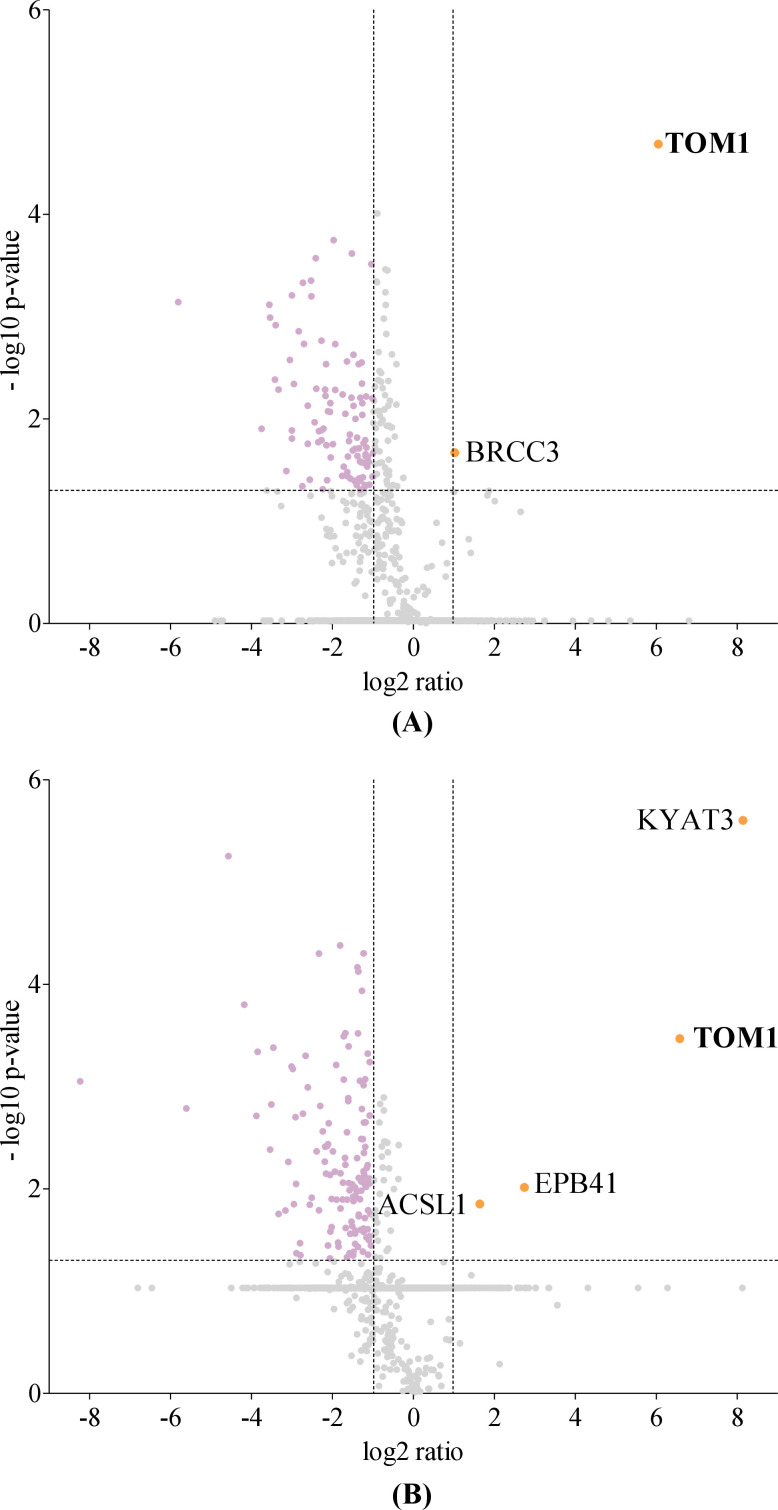
Differential abundant proteins in the supernatant proteome of PBMC after co-incubation with the HWF of A1 **(A)** or A2 **(B)** milk visualized in volcano plots. **(A)** Among the identified proteins, TOM1 and BRCC3 were significantly more abundant in the supernatant proteome of PBMC after co-incubation with the A1 HWF (orange), while 97 proteins were less abundant after the same co-incubation (purple). **(B)** Among the identified proteins, KYAT3, TOM1, EPB41 and ACSL1 were significantly more abundant (orange) in the PBMC supernatant proteome following co-incubation with the A2 HWF, whereas 132 proteins were less abundant under the same conditions (purple).

To elucidate the cellular mechanisms triggered by the inhibitory factor(s) in the HWFs of both milk variants, we analyzed the PBMC supernatant proteomes following co-incubation. The aim was to identify secreted proteins that reflect intracellular responses leading to the observed reduction in proliferation. Among the 2129 human proteins detected in the PBMC supernatant proteome, 99 showed significantly altered abundance after co-incubation with the A1 HWF: 97 proteins were decreased, and only two proteins were increased in abundance (**Figure 2A**). Co-incubation with the A2 HWF resulted in 136 proteins with significantly altered abundance, including 132 decreased and four increased proteins (**Figure 2B**). Thus, among all proteins detected in the PBMC supernatant proteome, only a small subset of proteins showed a statistically significant increase in abundance in response to co-incubation with the HWF of both A1 and A2 milk. In contrast to the analysis of proteins with higher abundance, the evaluation of proteins with reduced abundance revealed that a total of 176 proteins were significantly decreased in the PBMC supernatant proteome after co-incubation with HWF, with marked differences between A1 and A2 (**Supplementary Figure 4**; **Supplementary Table 5**).

### 3.4 TOM1 was significantly higher abundant following PBMC co-incubation with both the A1 and the A2 HWF

**Figure 3:**
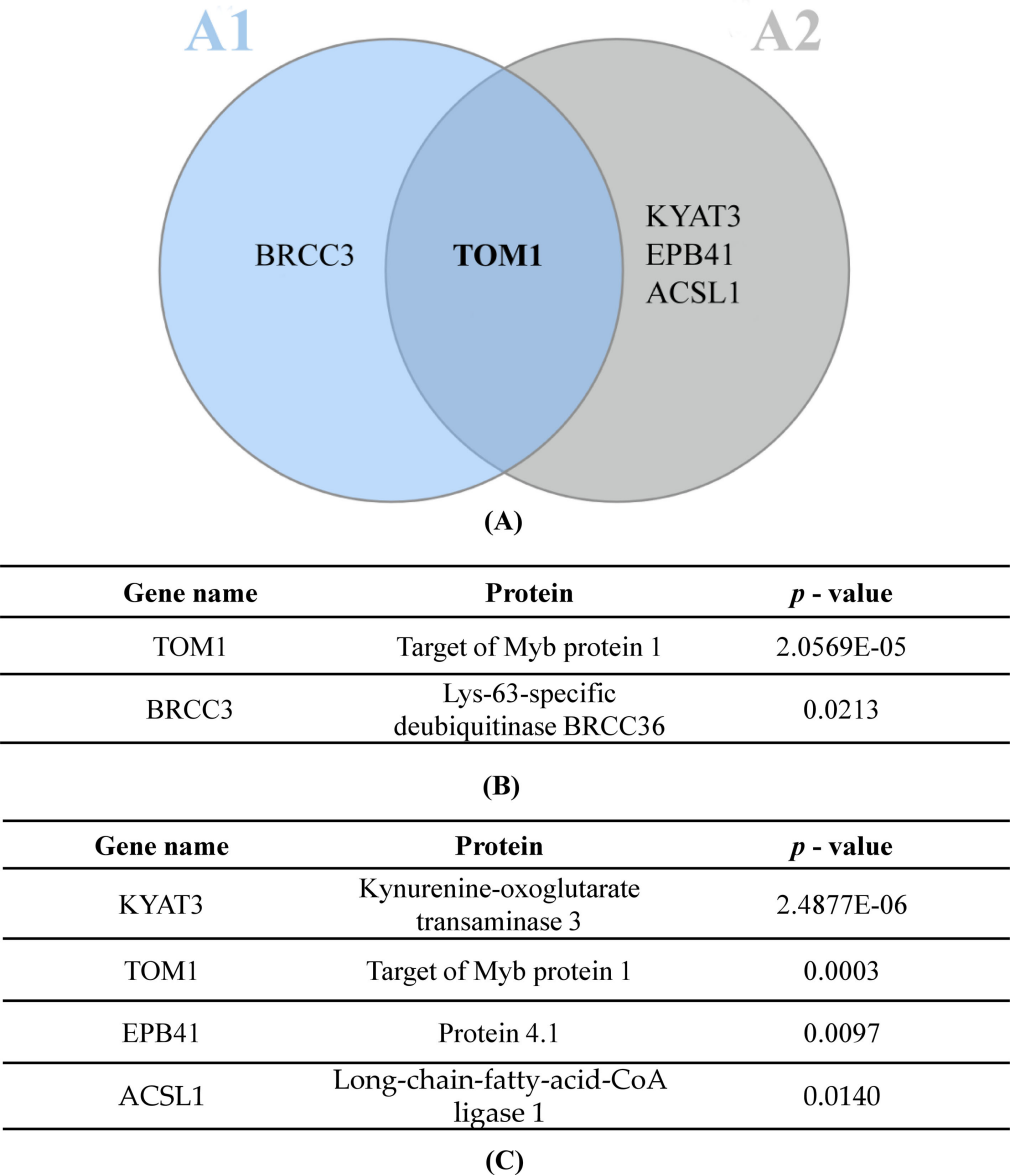
TOM1 was the only protein significantly more abundant in the PBMC supernatant proteome following co-incubation with HWFs of both milk variants. **(A)** Venn diagram visualizes the five proteins that were significantly more abundant in the PBMC supernatant proteome. After co-incubation with the A1 HWF (blue and dark blue), BRCC3 and TOM1 were significantly increased. Co-incubation with the A2 HWF (gray and dark blue) resulted in increased abundance of KYAT3, EPB41, ACSL1 and TOM1. Only TOM1 (dark blue) was significantly more abundant following co-incubation with both A1 and A2 HWF. **(B, C)** Listed were the proteins that were significantly more abundant in the supernatant proteome of ConA-stimulated PBMC after co-incubation with A1 HWF **(B)** and A2 HWF **(C)**. Columns indicate (1) gene names (2) corresponding protein names, and (3) statistical comparison of protein enrichment after co-incubation with either the A1 HWF **(B)** or the A2 HWF **(C)**, presented as p-values for each protein.

Particularly interesting to us were the few proteins with increased abundance, these were only two following co-incubation with A1 HWF and four following A2 HWF. Given the similar inhibitory effect of both HWFs on ConA-induced PBMC proliferation, we focused subsequent analyses on proteins that were consistently increased under both conditions, as potential mediators or indicators of the cellular pathways involved. Following co-incubation with the A1 HWF the protein Lys-63-specific deubiquitinase BRCC36 (BRCC3) was significantly more abundant in the PBMC supernatant proteome (**Figures 3A, B**). In contrast, co-incubation with the A2 HWF led to significantly increased secretion of Kynurenine-oxoglutarate transaminase 3 (KYAT3), Protein 4.1 (EPB41) and Long-chain-fatty-acid-CoA ligase 1 (ACSL1) (**Figures 3A, C**). Remarkably, only TOM1 was significantly increased in abundance under both conditions, with a 66.1-fold increase following A1 HWF (p = 0.00002) and a 95.4-fold increase following A2 HWF co-incubation (p = 0.0003) (**Figures 3A–C**). Importantly, TOM1 was consistently identified with multiple unique peptides, including sequences absent in the bovine homolog, such as SSPDLTGVVTIYEDLRR and VLELIPQIANEQLTEELLIVNDNLNNVFLR. The complete data of identified peptides of TOM1 is given in **Supplementary Table 3**.

The exclusive and significantly higher abundance of TOM1 in response to both milk variants, along with its pronounced fold change, directed subsequent investigations toward this protein as a potential mediator of the observed inhibitory effect.

### 3.5 Functional validation of TOM1 confirmed its inhibitory effect on polyclonally stimulated PBMC proliferation

**Figure 4:**
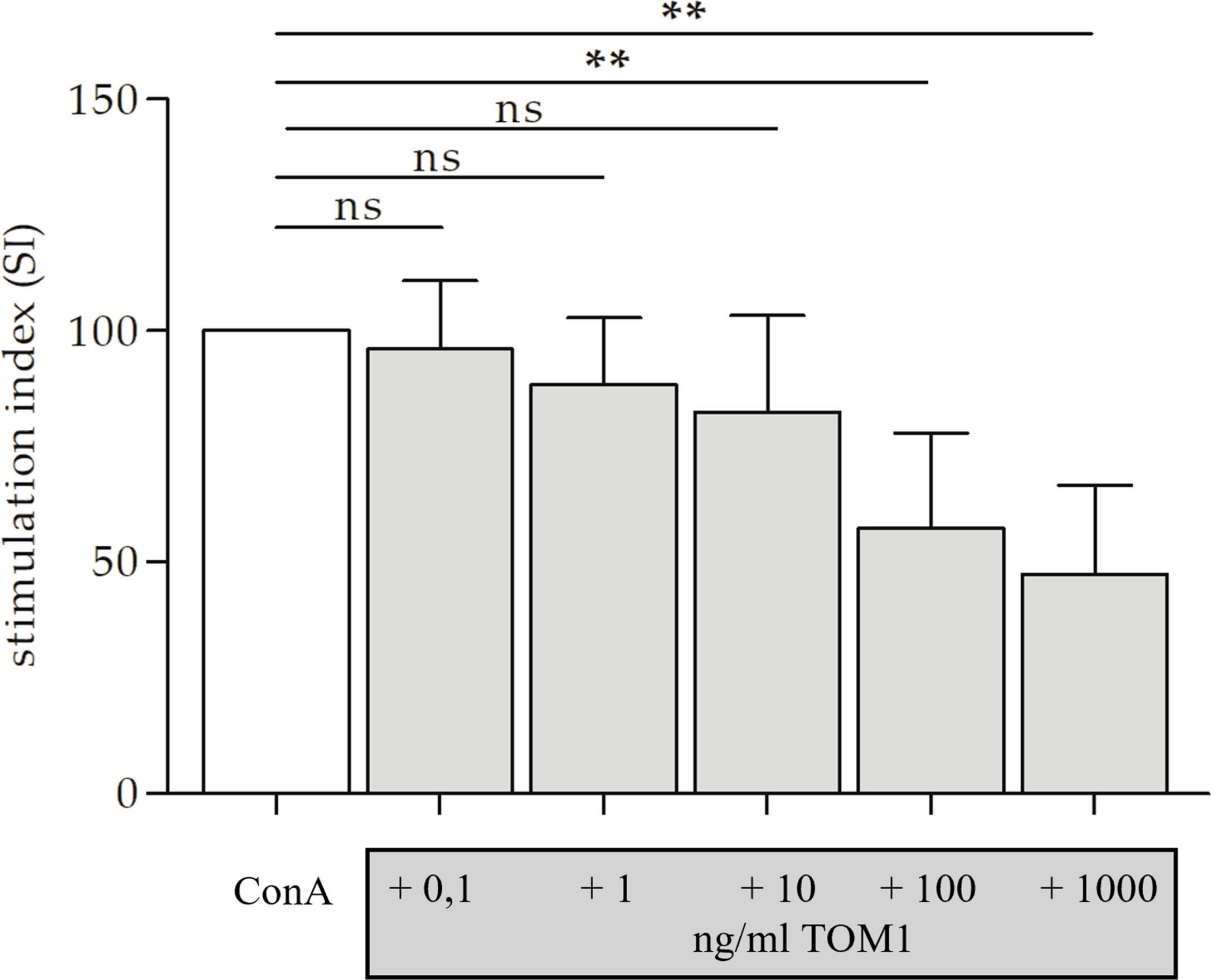
Effects of different concentrations of TOM1 on ConA-induced proliferation of human PBMC (n = 9). Bar plots represent stimulation indices of ConA-stimulated PBMCs following incubation with ascending concentrations of TOM1, n = 9. ConA-stimulated PBMCs served as control and were set to 100 (white), n = 9. The Shapiro–Wilk test was used to determine Gaussian distribution. As the control condition (ConA) showed no variance because of setting to 100, the Wilcoxon signed-rank test was applied for statistical significance; ns, p > 0.05; **, p < 0.005.

To determine whether TOM1 alone can exert an anti-proliferative effect and to further characterize its functional properties, polyclonally stimulated PBMC (n = 9) were co-incubated with purified TOM1 at several concentrations (**Figure 4**; gray columns). While TOM1 showed no measurable anti-proliferative effect at lower concentrations up to 10 ng/ml (**Figure 4**). From 100 ng/ml onwards, TOM1 significantly inhibited polyclonally stimulated proliferation (SI: 57.45 ± 20.47 SD; **, p < 0.005; **Figure 4**). At the highest tested concentration of 1000 ng/ml, TOM1 suppressed ConA-induced proliferation by more than 50% (SI: 47.40 ± 19.26 SD; **, p < 0.005; **Figure 4**).

### 3.6 PBMC developed an anti-inflammatory Immune-Response-Marker profile after co-incubation with TOM1

**Figure 5:**
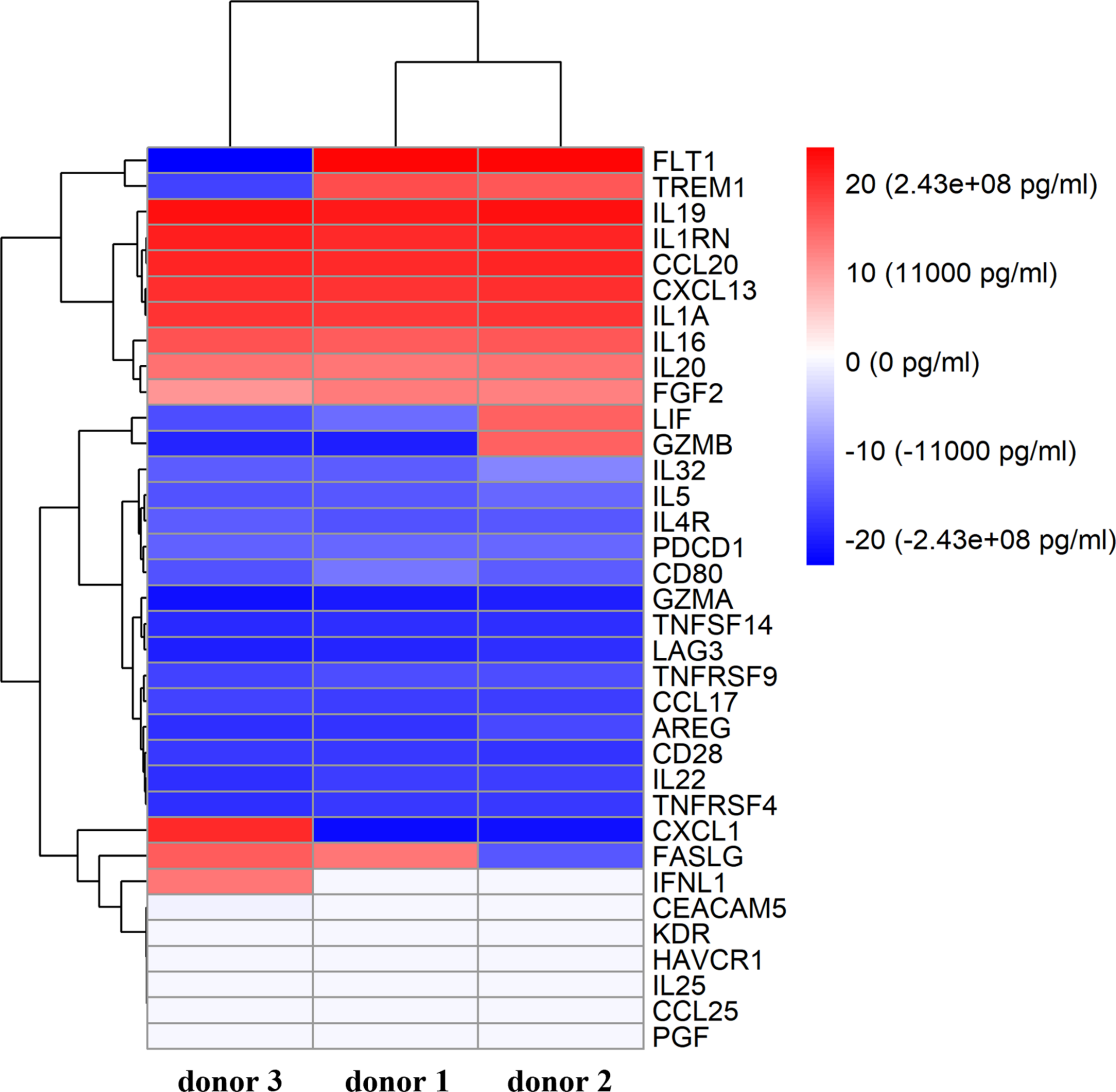
Secretion patterns of ConA-stimulated human PBMC after TOM1 co-incubation. Amount of secreted Immune-Response-Markers from human PBMC donors (n = 3) after co-incubation with ConA (1 µg/ml) and TOM1 (1 µg/ml) for 48h determined using the Olink Target 48 Immune Surveillance Panel. Background values (RPMI cell medium with ConA) were subtracted from the values after TOM1-incubation (ConA + TOM1) per donor, in order to analyze the protein amount exclusively induced by TOM1. Positive values (red) indicate TOM1-induced enhancement, negative values (blue) indicate lower abundance compared to ConA-stimulation alone.

To further determine the impact of TOM1 on stimulated PBMC, we performed an Olink proteomics experiment. ConA-stimulated (1 µg/ml) PBMC (n = 3) were co-incubated with purified TOM1 (1 µg/ml) for 48h and subsequently, changes in Immune-Response-Marker expression were analyzed in the PBMCs secretome using the Olink Target 48 Immune Surveillance Panel. After TOM1 co-incubation, a predominantly anti-inflammatory profile became apparent. Thus, anti-inflammatory proteins as Interleukin-1 receptor antagonist (IL1RN; also known as IL-1Ra) and Interleukin 19 (IL19) were highly enhanced by TOM1 (**Figure 5**; **Supplementary Table 6**). On the other hand, pro-inflammatory proteins like C-C motif chemokine ligand 17 (CCL17), Granzyme A (GZMA), Tumor necrosis factor receptor superfamily member 4 (TNFRSF4) as well as T-cell-specific surface glycoprotein CD28 (CD28) were reduced after TOM1 co-incubation (**Figure 5**; **Supplementary Table 6**).

## 4 Discussion

### 4.1 Summary of findings

In the present study, we investigated the effect of molecular weight fractions of cow’s milk on ConA-induced proliferation of primary human PBMC. This approach was based on our previous findings, that milk in general exerts an inhibitory effect on polyclonally stimulated human PBMC *in vitro* (11). Here, we identified the HWF (> 100 kDa) as the fraction carrying the anti-proliferative activity (**Figure 1**). An unbiased analysis of the PBMC supernatant proteome showed that TOM1 is the only protein that is consistently increased in abundance under co-incubation with both A1 and A2 HWF (**Figures 2**, **3**). Additionally, recombinant TOM1 was sufficient to reproduce an anti-proliferative effect on ConA-stimulated PBMC in a dose-dependent manner (**Figure 4**) and Immune-Response-Marker profiling supported an anti-inflammatory signature of TOM1 (**Figure 5**; **Supplementary Table 6**).

Until now, data describing the effects of milk fractionated by molecular weight on human ConA-stimulated PBMC *in vitro* do not exist. Under the conditions used here - 1 µg/mL ConA plus milk fraction at 1% v/v for 48 h - we observed a clear inhibition by the HWF and no effect of the LWF. Other studies using undivided milk at different time points and concentrations have reported divergent outcomes (27, 28), which is consistent with the idea that both milk preparation and stimulation conditions critically influence the measurable outcome.

The mitogen ConA binds to mannose residues and engages the T-cell receptor (29, 30). ConA is widely known as a T-cell stimulant (31, 32), although its exact mechanism of activation remains incompletely understood in humans (32). ConA-induced T-cell stimulation in mice involves CD28/CD80/CD86 co-stimulation and the herpesvirus entry mediator (32, 33). It is therefore plausible that the inhibitory effect of milk HWFs targets T-cell activation pathways.

### 4.2 Candidate proteins in the HWF-exposed supernatant proteome

To better understand which specific milk-derived components could be responsible for the observed effects, we focused on the immune response of PBMC during ConA-mediated cell activation in the presence of milk. We employed a proteomic approach and analyzed the supernatant proteome of human PBMC following ConA-stimulation and co-incubation with milk fractions. Using label-free LC-MS/MS, we generated the first high-resolution proteomic dataset of the PBMC supernatant proteome under these specific conditions, including co-incubation with A1 and A2 milk fractions. In our approach a total of 2640 human proteins were identified after 48 hours of incubation. Both milk variants induced significant changes in the supernatant proteome profiles of ConA-stimulated PBMC. Of the 2,640 identified proteins, 2,049 were common to both conditions.

Particularly notable were proteins that showed a significant increase in abundance under either condition. After A1 HWF exposure, BRCC3 and TOM1 were the two proteins found at elevated levels (**Figures 3A, B**). Following co-incubation with A2 HWF, the proteins KYAT3, EPB41, ACSL1, and TOM1 were significantly increased in the supernatant proteome of ConA-stimulated human PBMC (**Figures 3A, C**). These proteins were identified for the first time in the supernatant proteome of human PBMC under these conditions and in the context of a suppressive response during ConA-induced T-cell activation.

BRCC3 has previously been described exclusively in pro-inflammatory contexts in different cell types (34, 35). Even though there is evidence for BRCC3 to be expressed in human PBMC (26), its detection in an anti-proliferative experimental setting is unexpected and would represent a novel context-dependent function. The detection of KYAT3 in this context is interesting: to date, KYAT3 was not described as a secreted protein but there is evidence, that it can be expressed in human PBMC (26). KYAT3 is an enzyme that produces kynurenic acid (KYNA) (36), which signals via GPR35 and the aryl hydrocarbon receptor to promote anti-inflammatory cytokine profiles (37, 38), making it a plausible contributor. EPB41 and its murine homolog 4.1R act as negative regulators of T-cell activation via LAT phosphorylation, thereby suppressing the proliferation of both CD4 ^+^ and CD8 ^+^ T-cells *in vitro* and *in vivo* (39–41) and could contribute through a conserved mechanism. ACSL1, previously associated with pro-inflammatory programs in human monocytes and in murine CD4 ^+^ T-cells (42, 43), is here detected in a suppressive context and may represent a yet uncharacterized regulatory function. None of these four candidates was consistently increased in abundance under both A1 and A2 HWF, and we therefore treat them as secondary candidates meriting independent follow-up.

### 4.3 TOM1 as a promising candidate linked to the observed inhibitory response

Among the proteins increased in abundance in the HWF-exposed PBMC supernatant proteome, only TOM1 overlapped between the two milk variants (**Figure 2**,**Figure ** ). Given that A1 and A2 HWF inhibit ConA-stimulated PBMC proliferation to a comparable extent (**Figure 1**), the consistency of TOM1´s increased abundance across variants makes it the most compelling candidate mediator for the observed anti-proliferative effect. A recombinant-protein follow-up experiment confirmed that TOM1 alone reproduces the anti-proliferative effect on ConA-stimulated PBMC (**Figure 4**). TOM1 has not previously been described as a secreted protein of PBMC or T-cells, and its function in these cell types is uncharacterized.

In other human cell types, TOM1 is known to suppress inflammatory signaling. In HEK cells, intracellular TOM1 interacts with toll-interacting protein (TOLLIP) and adaptor molecules such as clathrin to inhibit downstream IL-1β signaling, thereby reducing activation of NF-κB and AP-1 (13), transcription factors essential for pro-inflammatory cytokines (15, 16). In addition, TOM1 promotes endosomal trafficking and lysosomal degradation of IL-1 receptor 1 (IL-1R1), the primary receptor for IL-1β, thus reducing receptor availability at the cell surface and dampening cellular responsiveness to IL-1β (14). Our identification of TOM1 in the supernatant proteome of ConA-stimulated PBMC under suppressive conditions therefore suggested a previously unrecognized role for TOM1 in immune regulation of PBMC, potentially mediating the common anti-proliferative effect induced by milk-derived HWFs.

To further substantiate the immunomodulatory role of TOM1, we performed multiplex profiling of Immune-Response-Markers (Olink) on PBMC supernatants following ConA-stimulation in the presence or absence of recombinant TOM1. The resulting secretome shifts strongly supported an anti-inflammatory mode of action. The most striking finding was a marked increase in IL1RN (Interleukin-1 receptor antagonist) secretion after TOM1 co-incubation (**Figure 5**, **Supplementary Table 6**). IL1RN is an endogenous competitive inhibitor of IL-1α and IL-1β signaling; by occupying IL-1R1 (Interleukin-1 receptor 1) without triggering downstream activation, it effectively neutralizes IL-1-driven inflammation (44). Recent mechanistic work demonstrates that even modest elevations in IL1RN can shift the IL-1α/β: IL1RN ratio toward a net anti-inflammatory state, because receptor occupancy by IL1RN prevents the assembly of the IL-1R1–IL-1RAcP signaling complex required for MyD88 recruitment and NF-κB activation (45). Notably, although TOM1-treated PBMC also secreted elevated IL-1α (**Figure 5**, **Supplementary Table 6**), the magnitude of IL1RN induction exceeded that of IL-1α, consistent with active counter-regulation rather than uncontrolled inflammation. TOM1 treatment also elevated IL-19, a member of the IL-10 cytokine family that signals through IL-20R1/IL-20R2 and exerts anti-inflammatory effects on monocytes and macrophages, including suppression of TNF-α and IL-6 (46). The higher expression of IL1RN and IL-19 suggests that TOM1 engages multiple, complementary immunoregulatory circuits.

In parallel, TOM1 exposure markedly reduced pro-inflammatory and cytotoxic effector signaling pathways. Thus, the release of granzyme A (GZMA) was highly reduced (**Figure 5**; **Supplementary Table 6**). These serine protease is one of the principal executors of cytotoxic T-lymphocyte and NK-cell killing (47). The downregulation implies that TOM1 blunts not only proliferative but also effector responses of activated lymphocytes - an observation that aligns with the diminished amount of T-cell co-stimulatory molecule CD28 detected in the samples (**Figure 5**; **Supplementary Table 6**), which is essential for T cell activation and expansion (48). The Tumor necrosis factor receptor superfamily member (TNFRSF) 4 also acts pro-inflammatorily by promoting the activation, proliferation and survival of T cells and enhancing the production of proinflammatory cytokines such as NF-κB (49). Also C-C motif chemokine ligand 17 (CLL17), a key molecule that mediates immune cell migration and inflammation (50), was substantially diminished (**Figure 5**; **Supplementary Table 6**).

In summary, we observed a dominant IL-1 axis counter-regulation via IL1RN with a simultaneous reduction of co-stimulatory and cytotoxic effector programs. Taken together, these data reinforce the hypothesis that TOM1 dampens ConA-induced PBMC activation predominantly through the IL-1 axis: intracellular TOM1 promotes IL-1R1 degradation, while its presence in culture - whether via secretion or experimental addition of recombinant protein - correlates with a pronounced shift in the extracellular milieu toward IL1RN dominance.

### 4.4 Mechanistic hypotheses for follow-up

We propose that TOM1 contributes to the anti-proliferative effect of milk HWFs via interference with IL-1β-associated signaling, possibly through the combination of intracellular promotion of IL-1R1 trafficking and degradation (13, 14). Direct tests would include quantification of NF-κB and AP-1 activity in PBMC or purified T-cells after co-incubation with HWFs and with recombinant TOM1, and neutralization experiments using anti-IL-1R1 or anti-IL1RN antibodies.

With respect to the upstream trigger of TOM1 secretion, public interaction data identified five overlapping interactors between human and bovine TOM1 (**Supplementary Figure 3**). Among these, Clathrin heavy chain 1 (CLTC; ~192 kDa) and Myosin VI (~150 kDa) are candidates on the basis of size (both fit the > 100 kDa fraction) and reported detection in bovine milk (24, 25). Furthermore, both interact with TOM1 in cellular pathways (51–53). Myosin VI complexes with TOM1 in autophagy-related membrane trafficking (51). Identifying which of these interactors are indeed present and functional in milk HWFs would provide important mechanistic insight into how TOM1 is activated, for example via endocytic trafficking or NF-κB regulatory pathways and could thereby help to explain the anti-inflammatory effects of TOM1 on PBMC.

### 4.5 Methodological limitations and future directions

Several limitations of the present study should be explicitly acknowledged. The first limitation concerns the pooling strategy employed at the milk-source level. Although milk was collected from three individual cows per genotype, the samples were subsequently pooled into a single genotype-level preparation, meaning that biological replication at the milk-source level is effectively n = 1 per genotype. Inter-cow variability can therefore not be assessed, and genotype-level inference is inherently constrained by this design. This approach was deliberately chosen to standardize cow-side conditions and to concentrate statistical power on the human PBMC-donor axis, which represents the primary source of biological variability in the present study. Future studies employing individual cow replicates would be needed to confirm that the observed immunomodulatory effects are consistent across a broader range of animals within each genotype. Second, the HWF and LWF are compositionally heterogeneous. The HWF contains intact proteins, casein micelles, milk fat globule membrane fragments and extracellular vesicles; the LWF contains peptides and soluble metabolites. No direct compositional profiling was performed. Third, fractions were added on a volume-matched (1% v/v) basis rather than on a protein-normalized basis, preserving the physiological stoichiometry between fraction and matrix within 1% milk. Fourth, donors were included in proliferation assays only if their PBMC exhibited a ≥ 15-fold proliferative response to ConA, which is appropriate as a quality-control criterion for this assay format but selects for ConA-responsive donors; generalizability to strongly hyporesponsive donors is not addressed here. Fifth, with respect to *in vivo* relevance, the use of undigested milk in co-incubation does not fully mirror digestion-dependent gastrointestinal conditions. Our previous work with *in vitro* digested milk (11) suggests that complete enzymatic breakdown of milk proteins in the HWFs would likely abolish their anti-proliferative activity. However, the buffering capacity of milk raises gastric pH, attenuating peptidase activity and restricting protein degradation in the stomach (54, 55). As a result, intact proteins may reach the small intestine, where they can be taken up by membranous epithelial cells prior to further enzymatic cleavage (56). Once absorbed and basolateral released by these cells (57), such proteins could interact with immune cells in the blood stream like PBMC and trigger inflammatory or allergic response (58). This effect might be amplified under conditions of increased intestinal permeability, such as in neonates or in the context of leaky gut, where PBMC could be exposed to substantially higher levels of milk-derived proteins than in healthy adults (59).

## 5 Conclusion

Milk exerted a strong inhibitory effect on polyclonally stimulated human PBMC, independent of the β-casein variant. The responsible factor was confined to the high molecular weight fraction (HWF). To characterize the cellular response, we applied an unbiased LC-MS/MS analysis of the PBMC supernatant proteome following co-incubation with A1 and A2 HWFs. Among 2640 identified human proteins, only a small subset showed differential abundance. Notably, only one protein, TOM1, was consistently increased after exposure to both A1 and A2 HWF. Subsequent functional assays demonstrated that TOM1 alone was capable of exerting a strong anti-proliferative effect on human PBMC. Furthermore, a predominantly anti-inflammatory profile of human PBMC became apparent in response to TOM1 co-incubation. Taken together, TOM1 represents a promising anti-inflammatory candidate linked to the observed anti-proliferative reaction of PBMC in response to the milk HWF fractions.

## Data Availability

The datasets presented in this study can be found in online repositories. The names of the repository/repositories and accession number(s) can be found below: https://www.ebi.ac.uk/pride/archive/, PXD066232.
