# Supplementary material for: Inhibitory impact of A1 and A2 cow’s milk on human peripheral blood mononuclear cells identifies TOM1 as a candidate anti-inflammatory mediator
Source: Front Immunol. 2026 Jun 10;17:1728935. doi: 10.3389/fimmu.2026.1728935 (PMC13290551; doi:10.3389/fimmu.2026.1728935)
Supplement: Supplementary file 1 [file DataSheet1.pdf]

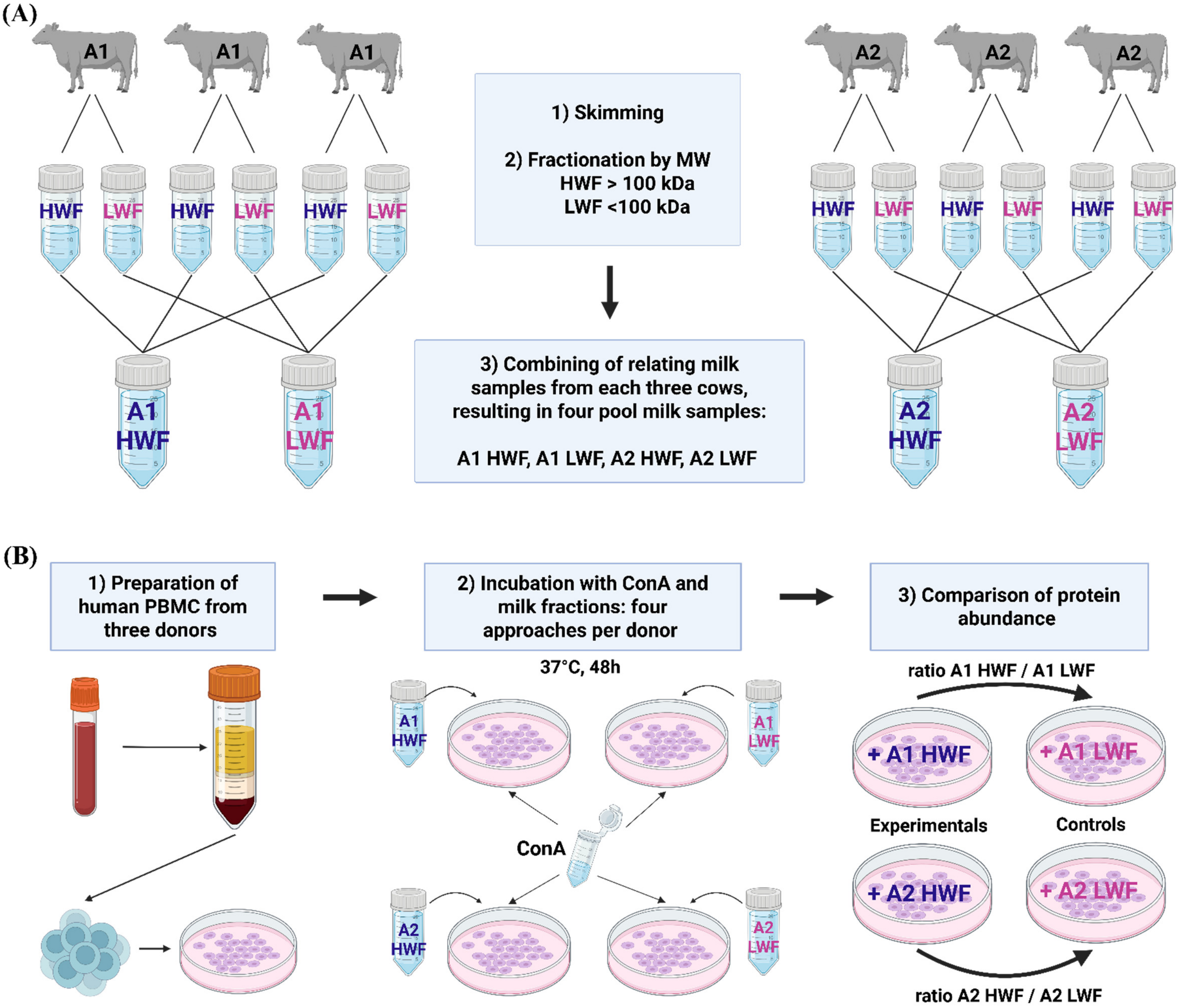

Figure S1. Workflow of (A) preparation of pool milk and (B) of human PBMC for proteomic measurement. Created with BioRender.com.

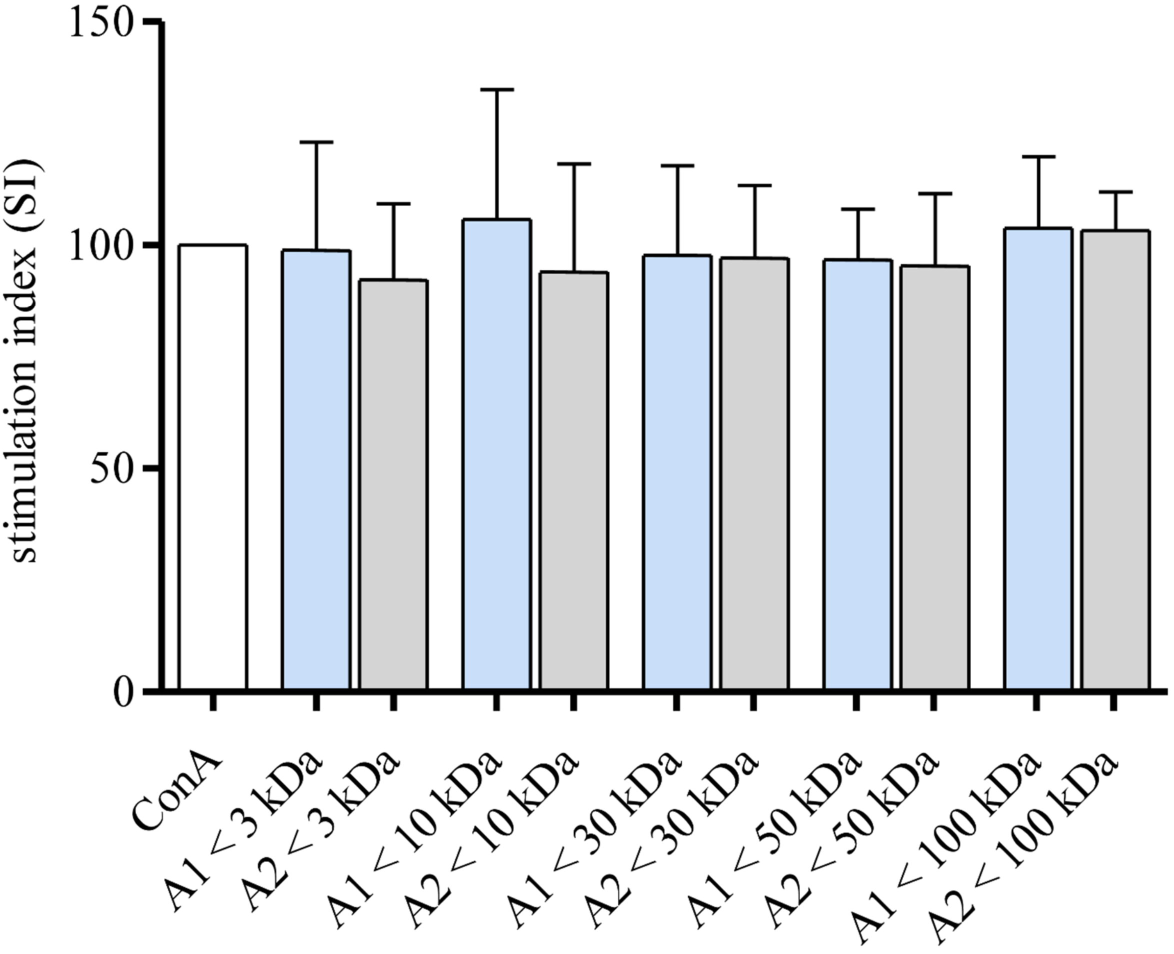

Figure S2. Effects of the four molecular weight fractions under 100 kDa (< 3 kDa, < 10 kDa, < 50 kDa and < 100 kDa) of A1 and A2 milk on ConA-induced proliferation of human PBMC (n = 5). Bar plots show the stimulation indices (SI) of polyclonally stimulated PBMC following incubation with milk fractions of A1 (blue) and A2 (grey) milk at a final concentration of 1%. ConA-stimulated PBMC without milk fractions served as a control and were arbitrarily set to 100 (white). All fractions < 100 kDa of both A1 and A2 milk had no inhibitory effect and no significant differences were found between the effects of A1 and A2 milk (ns = p > 0.05).

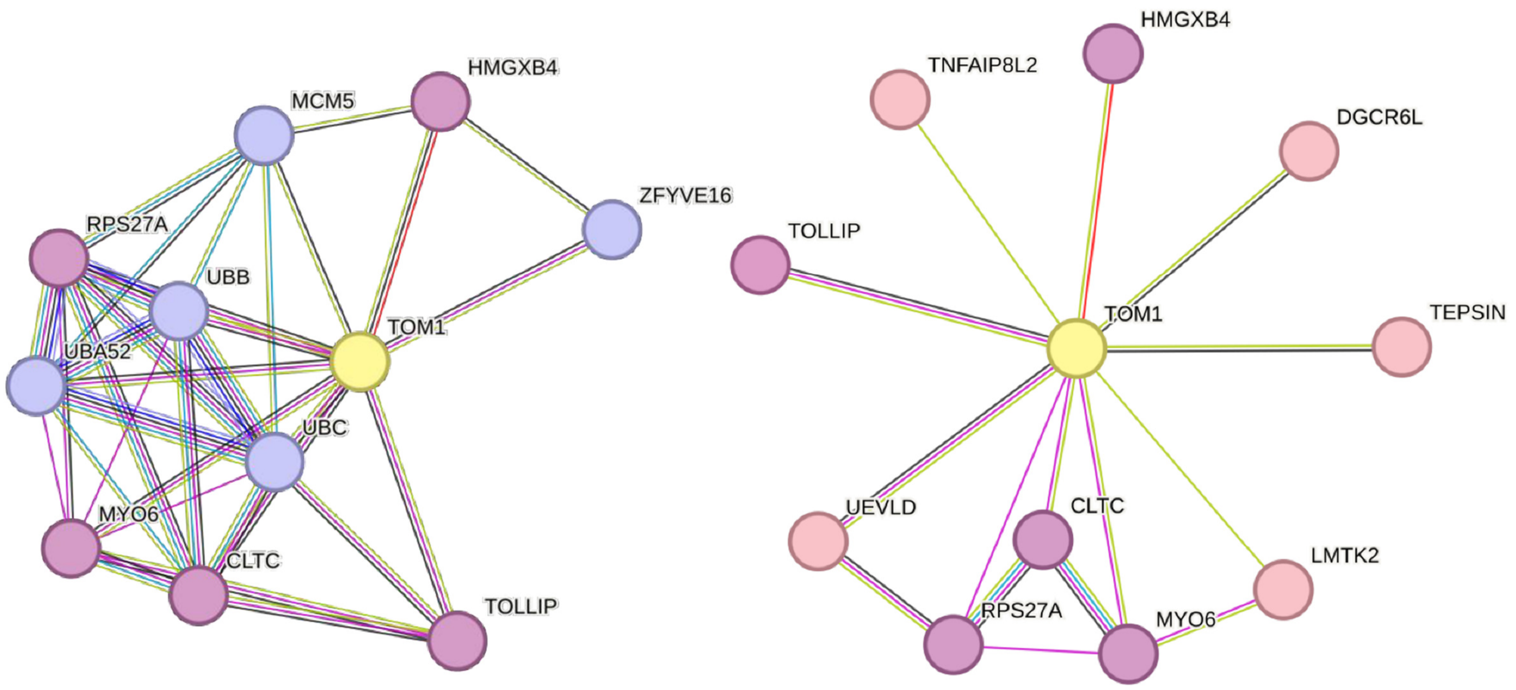

(A) **(B)**

Gene Name	Protein	Molecular weight (kDa) human / bovin
TOLLIP	Toll-interacting protein	30.28 / 30.08
CLTC	Clathrin heavy chain 1	191.62 / 191.59
MYO6	Myosin VI	149.69 / 149.62
RPS27A	Ubiquitin-ribosomal protein eS31 fusion protein	17.97 / 17.97
HMGXB4	HMG domain-containing protein 4	65.71 / 64.22

(C)

Figure S3. Known interacting proteins of TOM1. Protein-protein interactions of human **(A)** and bovine **(B)** TOM1 (yellow nodes) were visualized using the open-source software STRING version 12.0. The overlap between human and bovine TOM1 interactors (purple nodes) was of particular interest in the context of the hypothesis of interspecies protein interactions, compared to proteins interacting only with human (blue nodes) or bovine (red nodes) TOM1. The five overlapping proteins are listed in **(C)**, together with their molecular weights for the human and bovine versions.

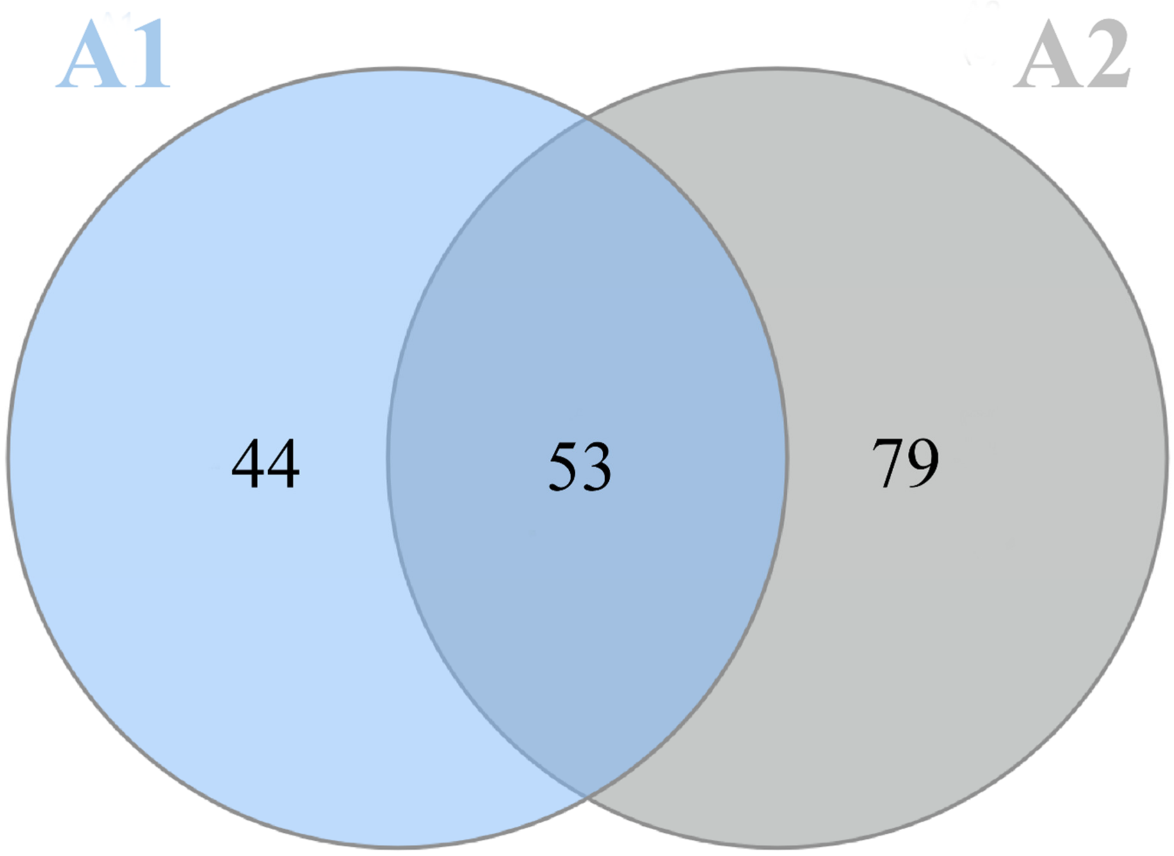

Figure S4. The proteins with reduced abundance in the PBMCs secretome are shown in a Venn diagram. In total, 176 proteins were significantly less abundant. After co-incubation with A1 HWF (blue and dark blue), 97 proteins were significantly decreased. Co-incubation with A2 HWF (grey and dark blue) resulted in a decrease of 132 proteins. Following co-incubation with both A1 and A2 HWF, 53 proteins had reduced abundance (dark blue). These results demonstrate that the overall changes were substantial, with clear differences between A1 and A2.
